# Atypical Teratoid Rhabdoid Tumor: A Possible Oriented Female Pathology?

**DOI:** 10.3389/fonc.2022.854437

**Published:** 2022-04-01

**Authors:** Cinzia Baiano, Rosa Della Monica, Raduan Ahmed Franca, Maria Laura Del Basso De Caro, Luigi Maria Cavallo, Lorenzo Chiariotti, Tamara Ius, Emmanuel Jouanneau, Teresa Somma

**Affiliations:** ^1^ Division of Neurosurgery, Università degli Studi di Napoli “Federico II”, Naples, Italy; ^2^ Department of Molecular Medicine and Medical Biotechnology, University of Naples “Federico II”, Naples, Italy; ^3^ Department of Pathology, University of Naples “Federico II”, Naples, Italy; ^4^ Division of Neurosurgery, Ospedale Santa Maria della Misericordia, Udine, Italy; ^5^ Division of Neurosurgery, Hopital Neurologique “Pierre Wertheimer”, Lyon, France

**Keywords:** sellar ATRT, endoscopic endonasal surgery, hystopathological features, multimodal approach, molecular biology

## Abstract

Atypical teratoid rhabdoid tumor is a rare lesion that occurs mainly in children can be supratentorial or infratentorial and it accounts for 1-2% of pediatric brain tumors and over 10% of central nervous system (CNS) tumors in infants, with a male preponderance up to 3 years of age, more than 50% of these occur in the cerebellum. In this report we describe four new cases of sellar AT/RTs underwent endoscopic endonasal approach and different adjuvant therapies. Our aim is to report the clinical, radiological and pathological features of these rare lesions, focusing on the possibility to perform an early diagnosis and appropriate therapeutic strategy.

## Introduction

Atypical teratoid rhabdoid tumor (ATRT) was first reported in 1981 by Biggs and colleagues as a new biologically aggressive central nervous system tumor presented in a three-month-old boy with unique clinical and morphological features (1). Afterwards, it was recognized as a separate tumor entity by the World Health Organization in 1993 (2), and was first included in the World Health Organization (WHO) Classification in 2000.

It is a rare lesion that occurs mainly in children can be supratentorial or infratentorial and it accounts for 1-2% of pediatric brain tumors and over 10% of central nervous system (CNS) tumors in infants, with a male preponderance up to 3 years of age, more than 50% of these occur in the cerebellum.

Despite aggressive treatments, ATRTs present poor prognosis, with a median survival less than 1 year. Currently, few reports described a survival of more than 2 years with the combination of radiation therapy and high-dose alkylator-based chemotherapy or by a multi-agent approach. Adult cases are rare and mostly supratentorial, and sellar localization is rare: there are 50 cases described in literature.

In this report we describe four new cases of sellar ATRTs underwent endoscopic endonasal approach and different adjuvant therapies. Our aim is to report the clinical, radiological, and pathological features of these rare lesions, focusing on the possibility to perform an early diagnosis and appropriate therapeutic strategy.

### Case 1

**Figure 1:**
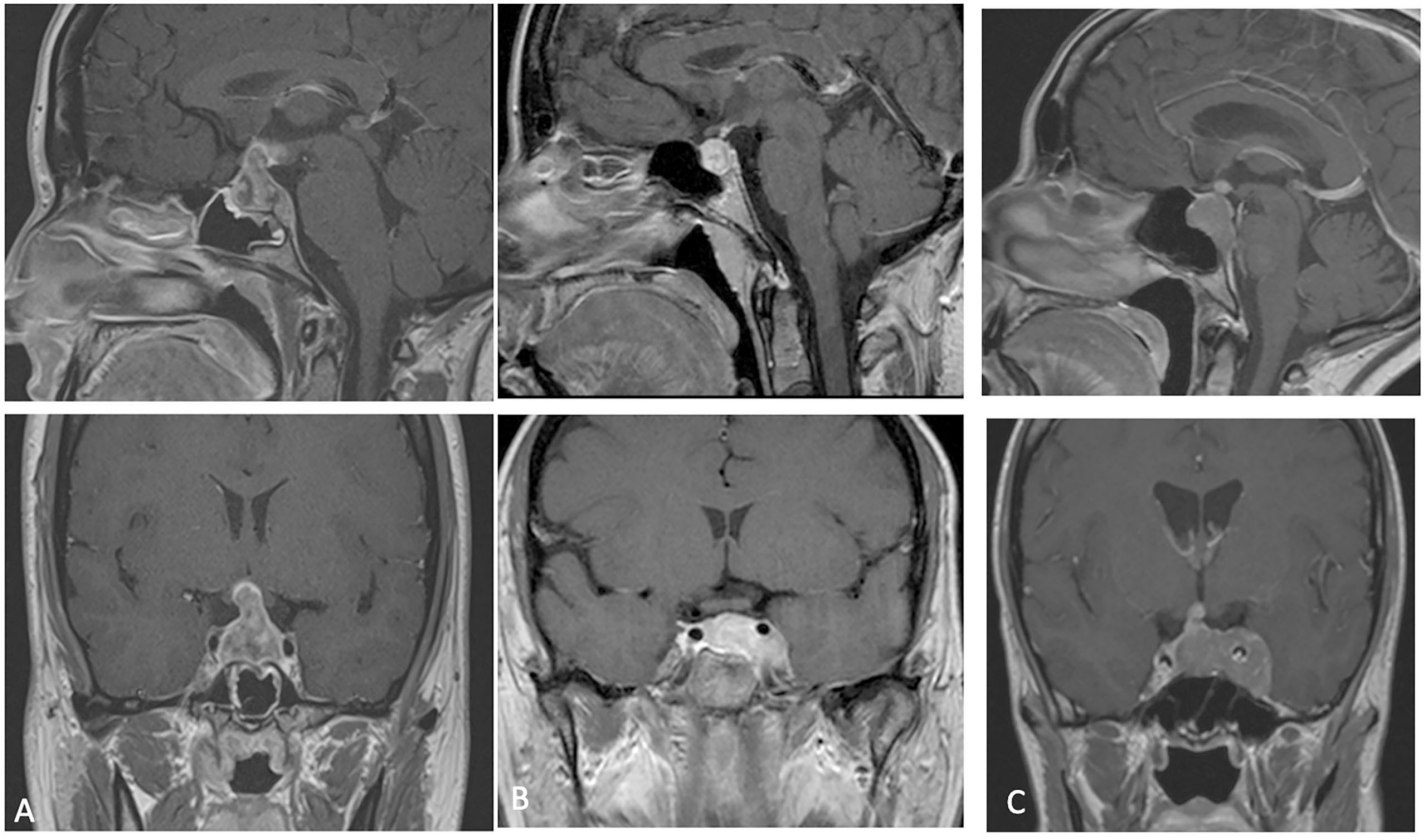
**(A–C)** preoperative MRI (cases 1-2-3) showing a lesion with heterogeneous post-contrast enhancement and intra supra and para-sellar extension.

A 32-year-old female not known to have any medical illnesses, mother of two children presented to our division, in January 2017, with a history of severe headache associated with double vision, vomiting, polyuria and polydipsia. The headache was progressive over 2 months, then became severe and associated with vomiting and double vision 10 days prior to presentation. Her neurological examination showed no neurological deficits apart from right sixth nerve palsy with pale optic disc and bitemporal hemianopia. The rest of examination was unremarkable. Hormone profiles showed a blood prolactin level slightly increased (36 ng/ml, n.v. 2.8-29.2). Preoperative magnetic resonance imaging (MRI) identified a large sellar mass with supra and para sellar extension (**Figure 1A**). Trans-sphenoidal approach and total tumor resection was performed. Postoperatively, the patient’s neurological status remained unchanged and was discharged without complication three days after the operation.

Thirteen days after resection, the patient was readmitted to our Clinic for headache associated with fever. She showed III and VI cranial nerves palsy with ptosis, ophthalmoplegia and amaurosis in OD associated with V1 hypoesthesia.

**Figure 2:**
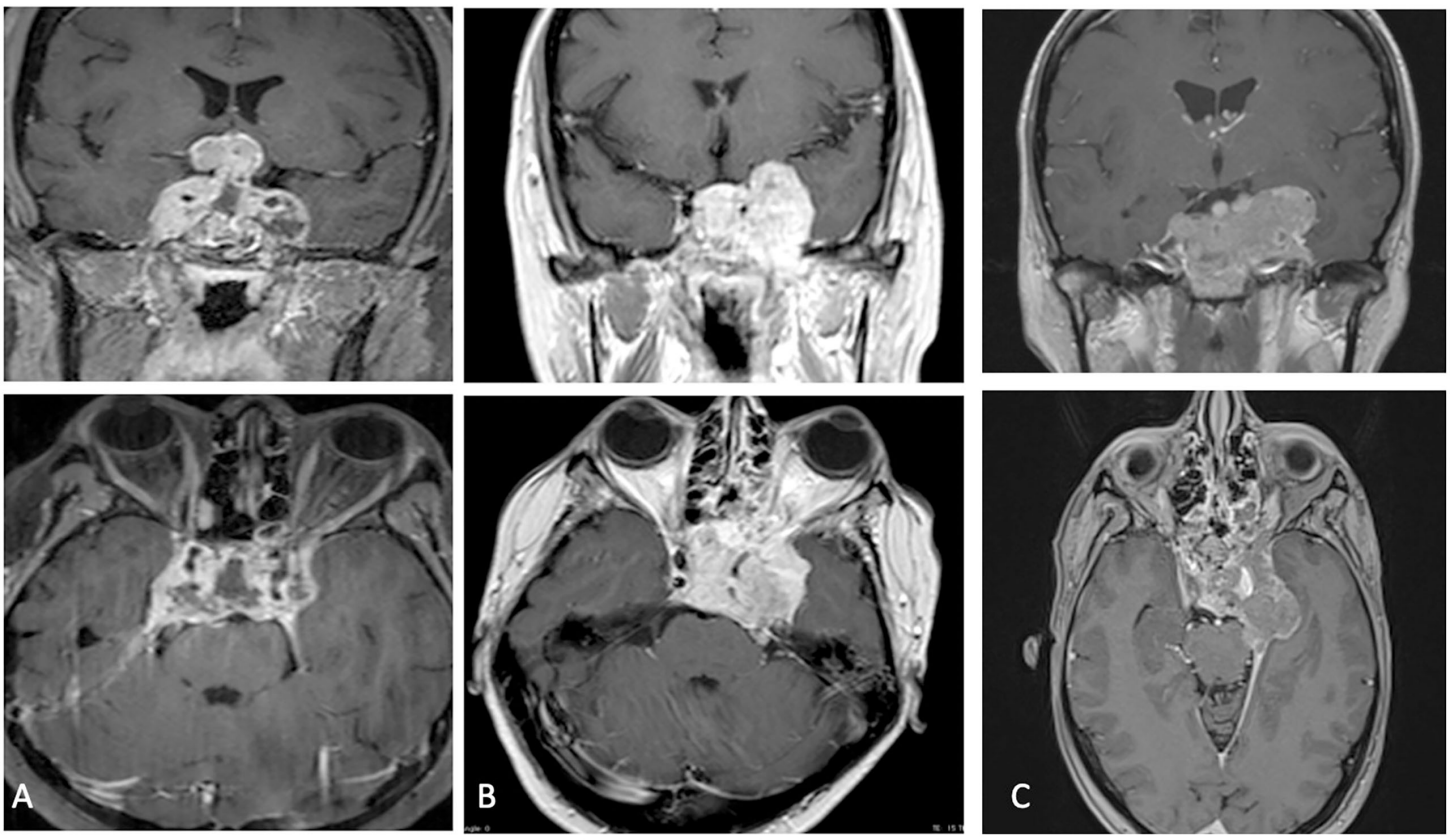
**(A–C)** Post-operative MRI at one month showing tumoral progression.

She underwent magnetic resonance imaging (MRI) of the *sella turcica* before and after an intravenous injection of contrast medium that showed the tumor regrowth in the original sellar location with extension to third ventricle (**Figure 2A**). It determined compression on the optic chiasma and involved both cavernous sinus with encasing the internal carotid arteries. Total body PET-CT scan was performed, and no pathognomonic signs were reveled, instead the evaluation of main serum tumor markers showed high serum level of chromogranin A. The histological examination demonstrated an atypical teratoid/rhabdoid tumor. She underwent the chemotherapy based on the use of bleomycin (three cycles), but she died before starting radiant therapy two months after diagnosis because of respiratory arrest due to involvement of larynx by the tumoral lesion.

### Case 2

A 40 years-old female was admitted to our Clinic, in July 2017, for intense retro-orbital headache, vomiting, double vision and eyelid ptosis in left eye. She underwent a brain and sellar MRI with and without contrast that showed an intra and left para-sellar lesion with heterogeneous contrast enhancement, left carotid artery encasement, and imprinting suprasellar cistern (**Figure 1B**). An ophthalmological evaluation detected a visual sensitivity of 11/10, and a Hess scheme introduced an incomplete third palsy and a sixth nerve palsy. The neurological examination at admission pointed out an incomplete ophthalmoplegia in left eye and double vision in all gaze directions and paresthesia in territory of innervation of V1. She underwent surgery with a gross total resection of the lesion and histological examination showed an atypical teratoid rhabdoid tumor. After that she practiced three cycles of chemotherapy with bleomycin. A postoperative brain MRI after one month indicated a progression of lesion (**Figure 2B**). A successive radiotherapy was programmed, but the patient died two months after diagnosis with cardiac arrest and before radiant treatment, not performed in reason of her poor performance status.

### Case 3

The third patient is a 41 years-old female with a clinical onset characterized by panhypopituitarism, hyperprolactinemia, and diabetes insipidus associated with extrinsic and intrinsic ophthalmoplegia in left eye, hospitalized in a different division of neurosurgery of Naples. Pre-operative brain and *sella turcica* MRI showed an intra, infra, supra, and left para-sellar lesion with heterogeneous contrast enhancement, left carotid artery encasement with extension to third ventricle and left tentorium of cerebellum infiltration (**Figure 1C**).

She underwent to endoscopic endonasal surgery obtaining the removal of the infra, intra, and suprasellar party: the first histological examination was not conclusive.

Then, considering progressive worsening clinical condition, a second histopathological examination was conducted at our Institute of Pathological Anatomy with a definitive diagnosis of ATRT with SMARCB1/INI1 inactivation. Unfortunately, the patient arrived at *exitus* for a cardiovascular arrest one month after surgery and without adjuvant treatment. The last neuroradiological examen of brain and sella turcica MRI highlighted an augmented extension of lesion mostly at level of left para-sellar region with mass effect on the left temporal lobe associated with left parapharyngeal space invasion (**Figure 2C**).

### Case 4

The fourth case deals with a 50-year-old female hospitalized and managed at Neurological Hospital “Pierre Wertheimer” of Lyon, in France. At clinical onset she presented intense headache, diabetes insipidus, hyperprolactinemia and anterior pituitary insufficiency, furthermore, she reported a visual acuity reduction associated with left intrinsic and extrinsic ophthalmoplegia. Brain and *sella turcica* MRI detected an intra, supra and left para-sellar lesion endowed with heterogeneous contrast enhancement and left carotid artery encasement. An endoscopic endonasal transsphenoidal approach was performed and a subtotal resection was achieved. The histological examination demonstrated an atypical teratoid/rhabdoid tumor with SMARCB1/INI1 loss.

Postoperative MRI study demonstrated the tumor progression with an increasing invasion of the lateral wall of the left cavernous sinus.

After a board discussion, total body PET-CT scan was performed which did not reveal tumor dissemination, and chemotherapy associated to proton therapy was started. A total of 6 cycles: 3 with VDC (vincristine, doxorubicin, cyclophosphamide) alternating with 3 cycles of IE (ifosfamide, etoposide) was used for chemotherapy. The treatment was well tolerated without significant acute toxicities and symptomatic relief was observed at the end of irradiation. The last control MRI demonstrated a volume reduction of the mass lesion. The patient is still alive and receives a regular follow-up at 18 months after surgery.

## Histopathological And Molecular Biology: Methodological Analysis Processing And Results

### Histopathology

**Figure 3:**
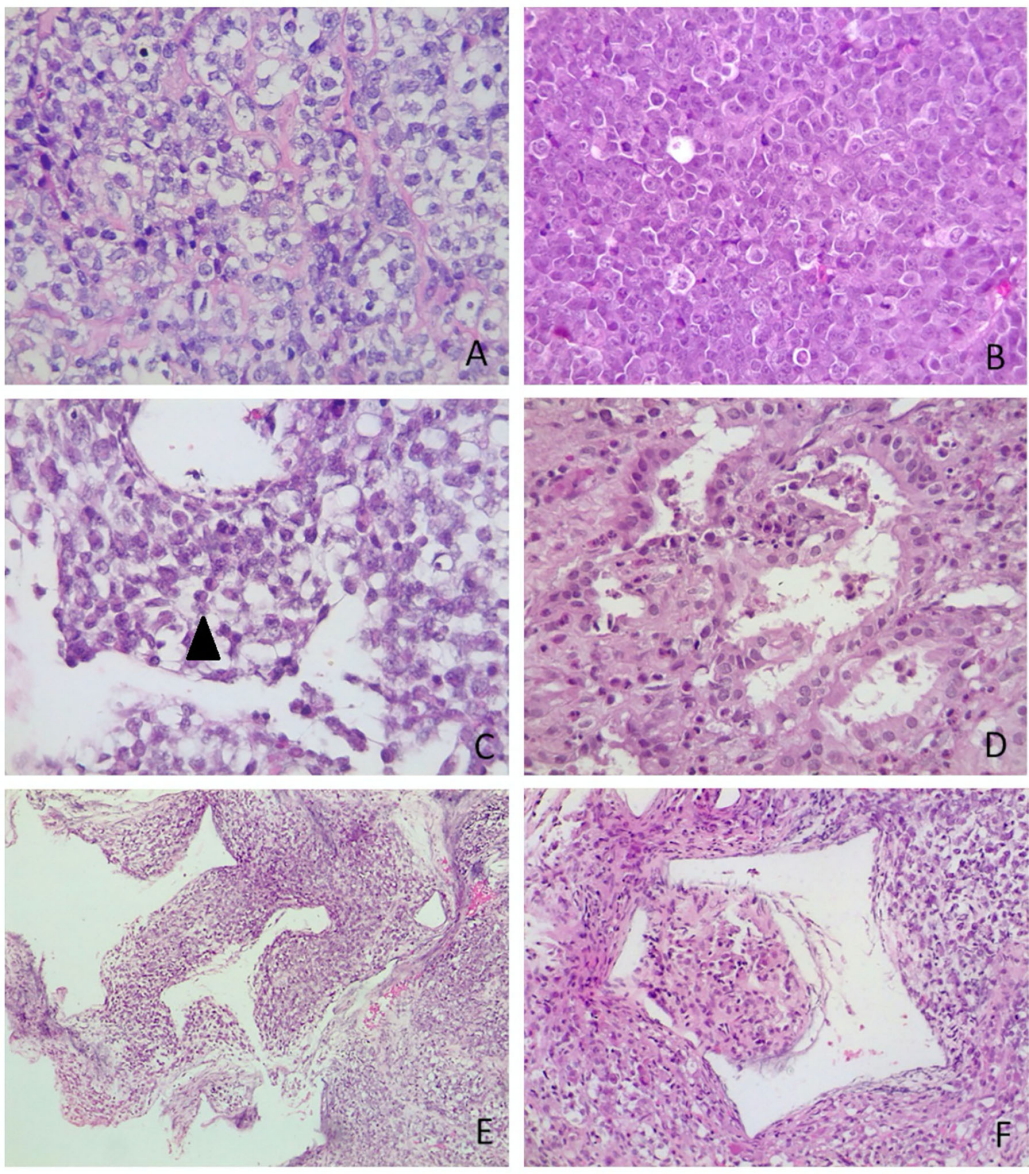
Microscopic appearance of sellar AT/RTs. **(A)** On microscopy, two out of three tumours were predominantly composed of diffuse proliferation of small to medium-sized cells with vesicular nuclei, prominent nucleoli and pale, clear or vacuolated cytoplasm. The variability of cytoplasmatic features created a “jumbled” appearance (case 3, *hematoxylin-eosin, original magnification 40x).* **(B)** In case 1 there was a more solid architectural growth. Tumour was premodominantly composed of large cells with eosinophilic cytoplasm, (case 1, *hematoxylin-eosin, original magnification 40x).* **(C)** Rhabdoid cells (arrowhead) were variably intermingled, having eccentric nuclei and eosinophilic intracytoplasmatic inclusions (case 2, *hematoxylin-eosin, original magnification 40x).* **(D)** Differentiation toward epithelial lineage: tumour cells were arranged in epithelial glandular structures, with lumen formation, lined by cylindric and ciliated epithelium (case 1, *hematoxylin-eosin, original magnification 40x).* **(E)** Thin-walled, branching haemangiopericytoma-like vessels (case 2, *hematoxylin-eosin, original magnification 10x).* **(F)** Botryoid appearance: polypoid nodule of tumour cells protruding into (case 2, *hematoxylin-eosin, original magnification 10x)*.

**Figure 4:**
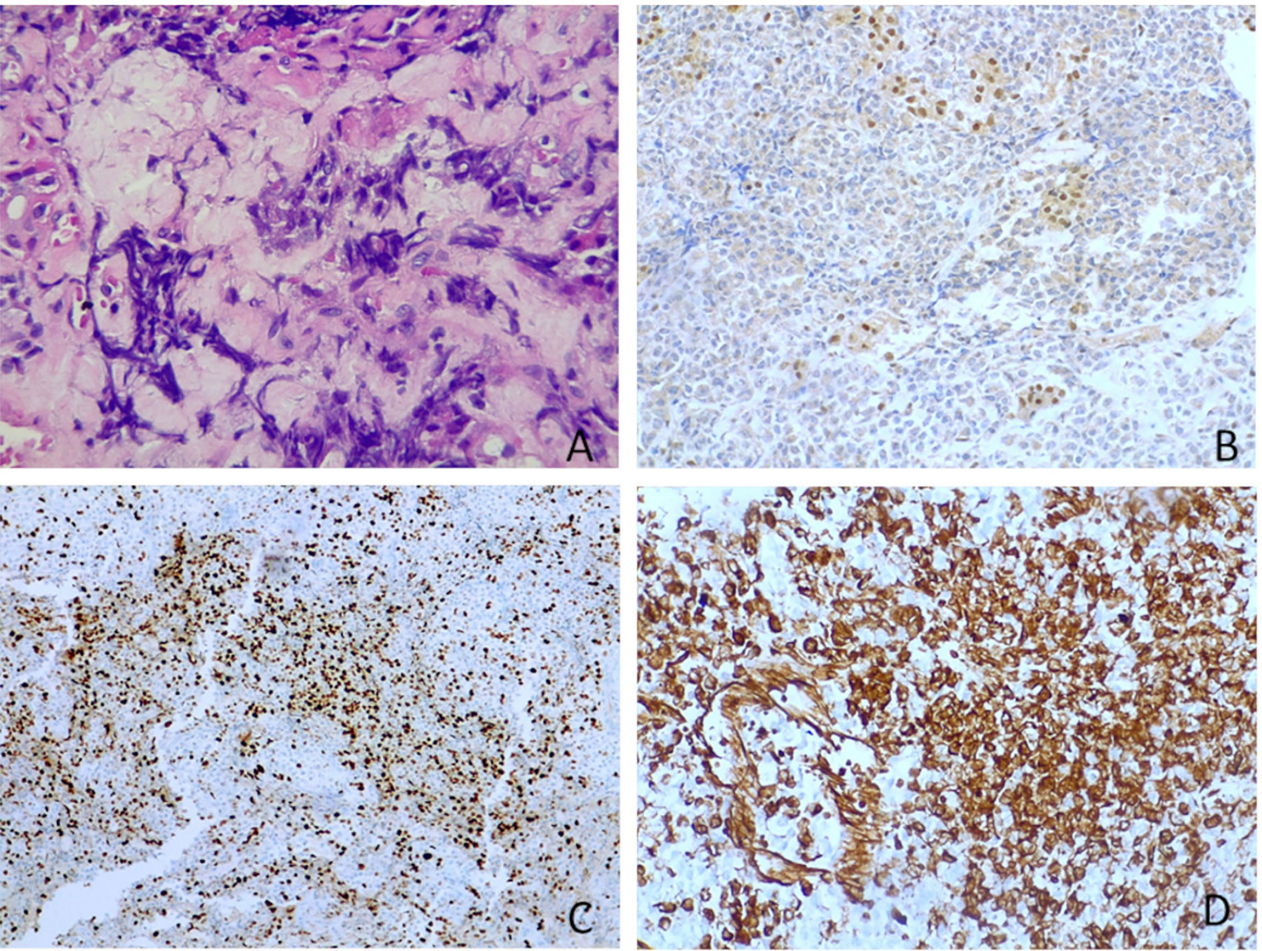
Other microscopic features and immunohistochemistry. **(A)** Myxoid stroma (case 3, *hematoxylin-eosin, original magnification 40x)*. **(B)** Tumour cells were negative for INI1, whereas adenohypophyseal cells were positive as an internal control (case 3, *immunoperoxidase staining, original magnification 20x).* **(C)** Ki67 was high in all ATRTs. In figure it was nearly 40% (case 3, *immunoperoxidase staining, original magnification 40x*). **(D)** Vimentin is positive in all tumours (case 3, *immunoperoxidase staining, original magnification 20x*).

Sections for morphology and immunohistochemistry were prepared from formalin-fixed paraffin-embebbed (FFPE) tissue specimens. Microscopic examination of hematoxylin and eosin-stained slides as well as immunohistochemistry were conducted for cases 1 to 4. Immunohistochemistry was performed with different antibodies, according to the main morphology-based differential diagnoses for each case. Sequent antibodies were applied: glial fibrillary acid protein (EP72Y, Rabbit monoclonal antibody, Cell Marque), vimentin (V9, mouse monoclonal antibody, Cell Marque), smooth muscle actin (1A4, mouse monoclonal antibody, Cell Marque), S100 (polyclonal, primary antibody, Ventana), epithelial membrane antigen (E29, mouse monoclonal primary antibody, Ventana), pancytokeratin (AE1/AE3, PCK26, primary antibody, Ventana), leukocyte common antigen (RP2/18, primary antibody, Ventana), CD99 (013, mouse monoclonal primary antibody, Ventana), CD1a (EP3622, rabbit monoclonal antibody, Cell Marque), synaptophysin (SP11, rabbit monoclonal primary antibody, Cell Marque), CD34 (QBEnd/10, primary antibody, Ventana), Ki67 (30-9, rabbit monoclonal antibody, Ventana) INI1 (25/BAF47, mouse antibody, BD Bioscences).Two out of three cases (case 2 and 3) were predominantly composed of diffuse proliferation of small to medium-sized cells with vesicular nuclei, prominent nucleoli and pale, clear, or vacuolated cytoplasm (**Figure 3A**). Conversely, case 1 showed a tumor populated by large cells with eosinophilic cytoplasm, more orderly arranged in a solid pattern (**Figure 3B**). In all three cases there was a variable number of rhabdoid cells with eosinophilic cytoplasm, containing a hyaline inclusion, and eccentric nuclei (**Figure 3C**). Only in case 1 there was a clear differentiation toward epithelial lineage: in some areas tumor cells were arranged in epithelial glandular structures, lined by cylindric and ciliated epithelium (**Figure 3D**). Also, Case 2 showed more compact areas populated by cells with high nuclear-cytoplasmatic ratio clustering predominantly around thin-walled, branching hemangiopericytoma-like vessels (**Figure 3E**). Focally, it can be observed a polypoid nodule of tumor cells protruding into a vascular lumen (botryoid appearance) (**Figure 3F**). In case 3 there was an obvious hyalinized to myxoid stroma (**Figure 4A**), that separated in some areas the neoplastic cells in ribbons and chords, giving the tumor a chordoma-like appearance. There were brisk mitotic activity and apoptotic figures. Immunohistochemistry revealed tumor cells were negative for INI1 in all cases (**Figure 4B**). Ki67 was high in all the samples, ranging from 40% (case 3 **Figure 4C**) to 90% (Case 1). Immunoreactivity for other antigens was detected (ATRT are typically polyphenotypic neoplasms) and results were reported in **Table 1**.

**Table 1:** Immunohistochemical profiles of three adult patients (case 1-4) with sellar region ATRT.

	GFAP	VIM	SMA	S100	EMA	CKPAN	LCA	CD99	CD1a	SYN	INI1	CD34
Case1	–	+	N/A	–	–	–	–	–	N/A	–	–	+ (F)
Case2	+ (F)	+	+	N/A	+ (F)	–	–	N/A	–	N/A	–	N/A
Case3	N/A	+	N/A	N/A	+ (F)	+ (F)	N/A	N/A	N/A	N/A	–	N/A
Case4	–	+	N/A	N/A	+(F)	–	N/A	–	–	N/A	–	N/A

GFAP, Gliofibrillary acid protein; VIM, vimentin; SMA, smooth muscle actin; S100, S100 protein; EMA, epithelial membrane antigen; CKPAN, pan-cytokeratin; LCA, leukocyte common antigen; SYN, synaptofisin; F, focal; N/A, not available.

### Molecular Biology

**Figure 5:**
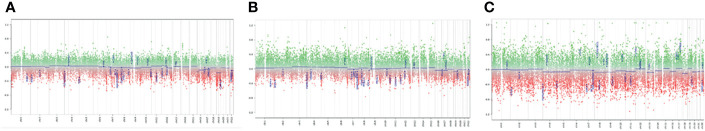
Copy Number Variations analysis: **(A)** Case 1; **(B)** Case 2; **(C)** Case C: The analyses of CNVs did not show genetic aberrations for the cases analyzed. Each panel indicated the analyses of CNV of the three tumor samples processed. We considered significant a gain of genetic materials if the score, obtained from ratio of case and control sample, was >0,4; as well, we considered significant a loss of genetic materials if the ratio score was <-0.4. We did not detect deletions for SMARCB1 (chr 22) in any of the three cases analyzed.

In order to verify whether methylome analysis could reinforce the diagnosis of ATRTs, we performed epigenomic characterization of 3 out of 4 of the above-described cases (cases 1 to 3). For DNA isolation from FFPE tissue, 10 × 10 μm sections were cut and DNA isolated using the Qiagen FFPE kit (according to manufacturer’s instructions). About 500 ng DNA was used for bisulfite conversion by the EZ DNA Methylation Kit (Zymo Research). Finally, the Infinium Methylation EPIC BeadChip Kit (Illumina) was used to quantify the methylation status of 850,000 CpG sites, on an iScan device (Illumina). Raw IDAT files were analyzed by bioinformatic tool previously published (3) (DKFZ, https://www.dkfz.de/de/index.html). Generated methylation data were compared with the Heidelberg brain tumor classifier to assign a subgroup score for the tumor compared with 91 different brain tumor entities (3). Belonging to a specific methylation class is assured by a “calibrated score” >0,9. However, values between 0,3 to 0,9 indicate similarity CNVs analyses did not show any alteration in INI copy number and no evident changes in other genomic regions were detected (**Figure 5**). CNVs were obtained by analyses of methylome raw data, comparing the intensity of methylated and unmethylated probes of sample analyzed with methylated and unmethylated probes of a control samples with a “flat “CNVs. The ratio obtained allows to identify gain or loss of specific genes and chromosome aberrations.

### DNA Methylation Profiling

CNVs analysis did not show any alteration in INI copy number and no evident changes in other genomic regions were detected (**Figure 5**).

Two out of three samples analyzed had a high calibrated score (> 0,9) for ATRT methylation class (0,98 and 0,97). The third case showed a similarity for the ATRT methylation class with a calibrated score =0,4. Subclass analysis showed that all 3 samples cluster into a specific epigenomic subclass: ATRT-MYC. This subclass includes ATRT with a supratentorial localization. All cases are associated with INI protein loss and activation of the MYC oncogene. These data confirm that methylome analysis is a useful tool to support the diagnosis of ATRT.

## Discussion

ATRT has been defined a “tumor of infancy” for its earliest onset and highest perinatal incidence (80.5%). In pediatric age ATRT can developed at supratentorial or infratentorial localization and it constitutes 1-2% of pediatric brain tumors and over 10% of central nervous system (CNS) tumors in infants, with a male preponderance up to 3 years of age, more than 50% of these occur in the cerebellum (4, 5). The most common clinical onset is characterized by intracranial hypertension. MRI features including intra-tumoral hemorrhage, peripherally localized cysts, high cellularity seen as low signal on T2 and ADC, heterogenous contrast enhancement similar to others malignant posterior fossa lesions such as medulloblastoma (6). Depending on molecular subgroup, the predominant location of ATRT in the brain is either supratentorial (ATRT–MYC) or infratentorial (ATRT-TYR). ATRT– SHH tumors can occur both supra and infratentorially (7).

Typical genetic alteration is the somatic mutation of SMARCB1 (formerly INI-1) or SMARCA4 (formerly BRG-1), retained immunohistochemical hallmarks of this tumor and demonstrated being an essential component of the ATP-dependent chromatin remodeling SWI/SNF complex, interacting with various pathways (p16-Rb pathway, Wnt-β-catenin pathway, sonic hedgehog signal pathway, polycomb pathway, MYCC, Aurora A) important for lineage specification, maintenance of stem cell pluripotency, and gene regulation (8).

Furthermore, this genetical condition is associated with the rhabdoid tumor predisposition syndrome (RTPS) characterized by a markedly increased risk of developing rhabdoid tumors commonly in the central nervous system more than 50% occur in the cerebellum, occurring predominantly in infants and children younger than age three years. Individuals with RTPS typically present synchronous tumors with aggressive clinical behavior before age 12; RTPS type 2 is associated with SMARCA4 germline mutation and with a worse prognosis (9). Then a test for identification of a germline heterozygous pathogenic variant in SMARCA4 or SMARCB1 by molecular genetic testing is indicated, at any age, in all cases of ATRT diagnosis (10–12).

We reported four novel cases of sellar ATRT in adult patients, and our series describe the classic phenotype of patients with this rare pathological entity: an adult female subject with acute symptoms of anterior and posterior pituitary insufficiency and ophthalmoplegia. As a matter of facts, sellar ATRT is almost exclusively diagnosticated in adult females and this suggest that this tumor is a clinicopathologically and genetically distinct variant of ATRT for its molecular and histopathologic features (13, 14).

On the base of a literature review, we found 50 cases of ATRTs reported before (12, 15–20); we have considered Articles that describe primitive sellar tumor cases in adults and were resulted ATRT with loss of INI, from 2000 to 2021 (**Table 2**).

**Table 2:** Literature review.

Study	Age	Sex	Surgical removal	Adjuvant treatment	Outcome
Kuge et al. (21)	32	F	STR	Craniospinal and focal radiation + Cisplatin, etoposide, and interferon	28 mo
Raisanen et al. (22)	20	F	STR	Radiation and chemotherapy (regimen not specified)	28 mo
Raisanen et al. (22)	31	F	STR	Radiation therapy (regimen not specified)	9 mo
Arita et al. (23)	56	F	STR	Radiosurgery: 17 Gy to periphery, 34 Gy to the center of residual tumor	23 mo
Las Heras et al. (24)	46	F	STR	NA	NA
Schneiderhan et al. (25)	61	F	STR	No adjuvant treatment	3 mo
Schneiderhan et al. (25)	57	F	GTR	Radiation therapy + Cisplatin and doxorubicin	6 mo
Chou et al. (26)	43	F	STR	Radiation therapy (regimen not specified)	2 we
Moretti et al. (27)	60	F	STR	Radiosurgery: 3400 cGy to center, 1700 cGy to periphery + Doxorubicin and vinorelbine; carboplatin and paclitaxel at progression	30 mo
Park et al. (28)	42	F	STR	Craniospinal irradiation at 3600 cGy with proton beam therapy and local boost 1800 cGy with cisplatin, followed by 11 rounds of multiagent chemotherapy: Cisplatin, doxorubicin, vincristine, etoposide, carboplatin, ifosfamide, cyclophosphamide	27 mo
Shitara et al. (29)	44	F	STR	Radiation therapy + 5 cycles of ICE chemotherapy	17 mo
Lev et al. (30)	36	F	STR	Proton beam radiosurgery + Temozolomide and a second line with Cytoxan, Adriamycin, and Vincristine and with Cisplatin and VP16	30 mo
Biswas et al. (31)	48	F	GTR	Vincristine, doxorubicin, cyclophosphamide, and ICE + craniospinal irradiation	2 mo
Larraín-Escandoín et al. (32)	43	F	STR	No adjuvant treatment	1 mo
Nobusawa et al. (33)	69	F	STR	Focal radiation + temozolomide	38 mo
Almalki et al. (16)	36	F	STR	vincristine with radiotherapy 60 Gy in 30 fractions, followed by 6 cycles of ICE	37 mo
Nakata et al. (13)	26	F	STR	local and spine radiotherapy with methotrexate followed by ICE	33 mo
Nakata et al. (13)	21	F	STR	local radiotherapy followed by ICE	35 mo
Nishikawa et al. (14)	42	F	STR	stereotactic radiosurgery 16 Gy then 14 Gy with temozolomide then radiotherapy 60 Gy with paclitaxel	11 mo
Johann et al. (34)	66	F	NA	NA	54 mo
Johann et al. (34)	20	F	NA	NA	120 mo
Johann et al. (34)	48	F	NA	NA	4 mo
Johann et al. (34)	46	F	NA	NA	Early after surgery
Barresi et al. (18)	59	F	STR	Fractionated radiotherapy	2 mo
Paolini et al. (15)	31	F	STR	No adjuvant therapy	2 mo
Paolini et al. (15)	36	F	STR	Radiation therapy and chemotherapy (regimen not specified)	22 mo
Paolini et al. (15)	46	F	STR	Not adjuvant therapy	Postoperative death
Paolini et al. (15)	47	F	STR	radiation and chemotherapy; 2000 cGy in 10 fractions and 3 agent chemotherapy (regimen not specified)	62 mo
Paolini et al. (15)	65	F	STR	Radiotherapy (5400 cGy in 30 fractions), chemotherapy (vincristine, cisplatin, doxorubicin, cyclophosphamide)	23 mo
Asmaro et al. (35)	62	F	STR	No adjuvant treatment	Postoperative death
Siddiqui et al. (36)	55	F	STR	No adjuvant treatment	6 weeks
Liu et al. (20)	43	F	STR	Radiation therapy and chemotherapy (regimen not specified)	4 mo
Liu et al. (20)	52	F	STR	NA	2 mo
Liu et al. (20)	50	F	GTR	NA	1 mo
Liu et al. (20)	29	F	STR	Radiation therapy (regimen not specified)	8 mo
Liu et al. (20)	80	F	STR	NA	1 mo
Peng et al. (37)	43	F	PR	Radiation therapy (regimen not specified)	4 mo
Peng et al. (37)	52	F	PR	No adjuvant treatment	2 mo
Peng et al. (37)	50	F	GTR	No adjuvant treatment	1 mo
Peng et al. (37)	29	F	PR	Radiation therapy and chemotherapy (regimen not specified)	8 mo
Peng et al. (37)	80	F	PR	No adjuvant treatment	1 mo

STR, subtotal resection; GTR, gross total resection; PR, partial resection; mo, months; N/A, not available.

The period of survival observed was included between 1 and 120 months and the most used therapeutic strategy was multimodal therapy based on trans-sphenoidal surgery, radio, and chemotherapy.

The transsphenoidal approach is the unique surgical technique utilized in the resection of these tumors and in all reported cases the lesion is characterized for fibrous consistency and absence of cleavage planes from the surrounding structures, so that, there are no total resection reported cases. Endoscopic endonasal technique is a valid instrument for neurovascular decompression and as this surgery provides adequate opportunity of obtaining tissue sample, crucial for continuing the integrated adjuvant therapies protocols (38).

Concerning adjuvant radiotherapy, the principal strategies were represented by fractionated radiation therapy and radiosurgery (gamma-knife). The protocols for adult ATRT recently developed are similar to the treatment protocols used for childhood ATRT: ICE protocol, anthracyclines, and cisplatin are most used chemotherapeutical agents (**Table 2**).

On microscopic examination ATRTs are known to be extremely heterogeneous tumors. In spite of their designation, the lesions are usually not composed largely of cells with rhabdoid features, but rather a jumble of medium to large cells with clear-appearing cytoplasm. At times, ATRTs can be predominantly “small cell” tumors, giving rise to a challenging differential diagnosis with other embryonal tumors, as well as with other “small round-blue-cell” neoplasms. Some authors (39, 40) identified three main histological patterns in pediatrics’ ATRTs: small round blue cell, mesenchymal/rhabdoid and epithelial.

Recently, three molecular subgroups of pediatrics ATRTs have been described: ATRT-TYR, ATRT-SHH, and ATRT-MYC based on cluster analysis of DNA methylation profiles. Recent studies (34, 41, 42) revealed morphological patterns in ATRTs are related to molecular subgroup status, reflecting the relationship between histopathological features, and underlying molecular alterations.

Recently, there are described three molecular subgroups of pediatrics ATRT: ATRT-TYR, ATRT-SHH, and ATRT-MYC based on cluster analysis of DNA methylation profiles. ATRT-TYR is characterized by infratentorial localization, younger age at diagnosis (<1year), and overexpression of the genes *TYR* and *MITF*. In the subgroup ATRT-MYC tumors are mostly supratentorial, older age at diagnosis (4-5 years), and the genes *MYC, HOTAIR*, and *HOX* are overexpressed. The subgroup ATRT-SHH tumor location may be infratentorial or supratentorial, diagnosis is between ages two and five years, and sonic hedgehog pathway genes are overexpressed (34, 42, 43). Epigenetic analyses revealed that the ATRT-TYR subgroup, and to a lesser extent also the ATRT-SHH subgroup, but not the ATRT- MYC subgroup, were characterized by a hypermethylated genome. These different molecular subgroups are in part due to a different distribution of partially methylated domains (PMD), domains of disordered methylation, sometimes covering up to 30% of the total genome. PMDs are almost absent in ATRT-TYR cases, shows a variable distribution in ATRT-SHH cases, and are most prevalent in ATRT- MYC cases where they occupied up to 36% of the genome (42, 44). Finally, gender distributions between the subgroups did not show significant differences. Sellar region ATRT is a clinically distinct group demonstrating epigenetic similarities with pediatric ATRTs of the ATRT-MYC subgroup (overexpression of the *MYC* oncogene) (34). In sellar ATRT HOX genes are hyper expressed similarly ATRT-MYC sub-molecular group (42). Interestingly, in a cohort of patients described in a previous study (41), a group identified as 2B ATRT seemed to have an increasing predominance in older individuals. However, we were no able to find specific signatures or profiles in the here reported case series that align with observations of the subgroups described in Torchia et al.

In adult patients, ATRT has a predilection for midline structures, particularly the pineal and pituitary glands, recognized as a source of neural stem cells in adults (45). Sellar and suprasellar region is a preferential site of adult ATRT, as a matter of facts, no ATRT in this site has been found in pediatric population; whereas a we have found in literature an only one case of cavernous sinus ATRT detected in a 18-month-old girl (21, 23, 25, 46, 47).

Concerning neuroradiological features of sellar ATRT we found intra, supra and para-sellar lesions with heterogenous contrast enhancement without distinctive radiological signs respect to a pituitary adenoma, whereas the postoperative MRI after two weeks showed a massive tumor progression and neurovascular midline surrounding structures infiltration compatibles with high grade lesions.

In our series there are two patients arrived to *exitus* three months after surgery during chemotherapy and radiotherapy, one patient died before adjuvant treatment after a month, and one patient is still alive today with a protocol VCD/IE and proton therapy after 18 months after surgery. Conforming literature data, these patients are female and with poor prognosis.

Common genetic feature in our series is the loss of SMARCB1 (also known as hSNF5/INI1), a core member of the SWI/SNF chromatin remodeling complex. It’s possible of detecting this genetic alteration also in others midline tumors such as poorly differentiated chordomas, and this genetic alteration changes the prognosis and make it unexpectedly unfavorable compared to classical chordomas with SMARCB1 retained (48).

Regarding these molecular alterations, the future therapeutical perspectives are oriented to molecular pathways modulation and several trials are ongoing. The first phase I/II trials employing agents targeting molecular is started for Aurora Kinase A Inhibitors, Hedgehog/GLI inhibitors, and EZH2 inhibitor (43, 49).

This tumor represents a model disease for malignancies not purely based on genomic mutational events but also on epigenetic alterations so that others targeted trials are required for detecting other biological treatment combined with epigenetic inhibitors, immune therapy as well as conventional chemotherapy with RT to refine therapeutic approaches to ATRTs RT (50).

## Conclusions

Sellar ATRTs are rare lesions for histopathology and localization with poor prognosis.

Endoscopic endonasal approach is an instrument for early diagnosis and treatment of these lesions due to absence of clinical distinctive features.

Multimodality treatment approach is most commonly used in the management of this pathology so that prognosis improvement is possible by the identification of effective adjuvant therapies to control relapse and progression.

## Data Availability Statement

The original contributions presented in the study are included in the article/supplementary material. Further inquiries can be directed to the corresponding author.

## Ethics Statement

This study was approved by the Institutional Review Board of the School of Medicine of Università degli Studi di Napoli “Federico II”, which waived the necessity for informed consent due to the retrospective nature of the study. Written informed consent was obtained from the patients prior than any invasive clinico-diagnostic and surgical procedure; indeed, it was obtained for the eventual publication—for scientific purpose—of any patient records/information anonymously. The patients/participants provided their written informed consent to participate in this study. Written informed consent was obtained from the individual(s) for the publication of any potentially identifiable images or data included in this article.

## Author Contributions

TS and CB: conception and design. CB, RD, and RF: acquisition of data. MB, LMC, EJ, LC, and TI: supervision and validation. CB, RD, RF, and TS: writing original draft and review and editing. All authors have read and agreed to the published version of the manuscript.

## Conflict of Interest

The authors declare that the research was conducted in the absence of any commercial or financial relationships that could be construed as a potential conflict of interest.

## Publisher’s Note

All claims expressed in this article are solely those of the authors and do not necessarily represent those of their affiliated organizations, or those of the publisher, the editors and the reviewers. Any product that may be evaluated in this article, or claim that may be made by its manufacturer, is not guaranteed or endorsed by the publisher.
